# Haemoglobin A1c-based screening for prediabetes and diabetes mellitus: a multi-center study in Croatian adult population

**DOI:** 10.11613/BM.2022.010903

**Published:** 2021-12-15

**Authors:** Ivana Lapić, Dunja Rogić, Nora Nikolac Gabaj, Katarina Kajić, Nena Peran, Lada Surjan, Anamarija Đuras, Valentina Cesar Kocijan, Nada Bilopavlović, Fran Smaić, Mario Štefanović, Ivanka Ostroški, Leida Tandara, Milena Krnjaić-Tadijanović, Ivan Gornik, Hrvoje Pintarić, Daniela Marasović Krstulović, Blaženka Miškić, Dario Rahelić

**Affiliations:** 1 Department of Laboratory Diagnostics, University Hospital Center Zagreb, Zagreb, Croatia; 2 Faculty of Pharmacy and Biochemistry, University of Zagreb, Zagreb, Croatia; 3 Department for Clinical Chemistry, University Clinical Hospital Sestre milosrdnice, Zagreb, Croatia; 4 Laboratory for Medical Biochemistry, General Hospital Bjelovar, Bjelovar, Croatia; 5 Department of Laboratory Diagnostics, General Hospital of Šibenik-Knin County, Šibenik, Croatia; 6 Laboratory for Medical Biochemistry, General Hospital Varaždin, Varaždin, Croatia; 7 Department of Medical Laboratory Diagnostics, University Hospital Center Split, Split, Croatia; 8 Department of Laboratory Diagnostics, General Hospital ‘Dr Josip Benčević’, Slavonski Brod, Croatia; 9 Department of Emergency Medicine, University Hospital Center Zagreb, Zagreb, Croatia; 10 Emergency Department, University Clinical Hospital Sestre milosrdnice, Zagreb, Croatia; 11 School of Dental Medicine, University of Zagreb, Zagreb, Croatia; 12 Internal Clinic, University Hospital Center Split, Split, Croatia; 13 Department for Internal Medicine, General Hospital ‘Dr Josip Benčević’, Slavonski Brod, Croatia; 14 Department of Diabetes and Endocrinology, Vuk Vrhovac University Clinic for Diabetes, Endocrinology and Metabolic Diseases, Merkur University Hospital, Zagreb, Croatia

**Keywords:** haemoglobin A1c, prediabetes, diabetes mellitus, prevalence

## Abstract

**Introduction:**

Based on the hypothesis that there is a substantial rate of adults with prediabetes and undiagnosed diabetes mellitus (DM), our aim was to perform haemoglobin A1c (HbA_1c_)-based screening in a cohort of Croatian adults and estimate the prevalence of prediabetes and undiagnosed DM according to American Diabetes Association criteria.

**Materials and methods:**

This multi-center, cross-sectional study performed in six Croatian hospitals included 5527 patients aged 40 to 70 years admitted to the Emergency Department or undergoing a primary care check-up. Haemoglobin A1c was measured from leftover whole blood samples using the enzymatic method on either Alinity c or Architect c-series analyser (Abbott Laboratories, Chicago, USA). Haemoglobin A1c between 39-47 mmol/mol was classified as prediabetes, while ≥ 48 mmol/mol as undiagnosed DM.

**Results:**

After exclusion of 435 patients with known DM, the final cohort included 5092 patients (median age 57; 56% males). A total of 882 (17.3%) patients had HbA_1c_ values between 39 and 47 mmol/mol. There were 214 (4.2%) patients with HbA_1c_ ≥ 48 mmol/mol. Prediabetes prevalence ranged from 14.2% to 20.5%, while undiagnosed DM from 3.3% to 7.3%, with statistically significant differences among settings (P < 0.001). Age-stratified analysis showed that prediabetes and undiagnosed DM prevalence increase with age (P < 0.001), being 25.4% and 5.8%, respectively, in patients aged 60 to 70 years.

**Conclusion:**

Underlying impairment of glucose metabolism was identified in about one in five adults, with significant number of patients with already overt DM. These results should serve as a starting point for further steps directed towards promotion of preventive measures for DM in Croatia.

## Introduction

Diabetes mellitus (DM) is the most common chronic metabolic disorder characterized by hyperglycaemia due to impaired insulin secretion or increased insulin resistance. It has a constantly increasing global prevalence, with estimated 463 million adults aged 40 to 79 affected worldwide, which makes it the major ongoing healthcare epidemic (*1*). Development of type 2 DM (T2DM) is preceded by prediabetes, a silent condition characterized by somewhat elevated fasting glucose and/or impaired glucose tolerance (IGT). It is of utter importance to note that prediabetes is a reversible disorder and that its conversion to T2DM and the development of associated complications can be easily prevented by timely recognition and introduction of simple lifestyle changes. Therefore, simple, large-scale screening laboratory methods that effectively identify asymptomatic individuals with prediabetes are strongly advocated. The American Diabetes Association (ADA) recommends screening of all adult individuals older than 45 years and without known DM every 3 years (*2*). Proposed screening methods considered equally appropriate are either fasting plasma glucose (FPG), oral glucose tolerance test (oGTT) or measurement of haemoglobin A1c (HbA_1c_) (*1*, *2*). Haemoglobin A1c concentrations reflect the average blood glucose concentrations within the erythrocytes’ lifespan and can serve as a long-term indicator of glucose metabolism regulation (*3*). The ADA criteria define that HbA_1c_ values between 39 and 47 mmol/mol indicate prediabetes, while HbA_1c_ concentrations equal to or above 48 mmol/mol are considered as manifested DM (*2*).

Early recognition of prediabetes through HbA_1c_-based screening is being increasingly implemented worldwide, yielding remarkably high prevalence, as seen in a Swiss cohort of healthy adults where 30.9% of participants were identified with prediabetes (*4*). According to the data from the Croatian registry, there are around 310 thousand registered DM patients in Croatia, resulting in a 6.8% prevalence in adult population. However, the true prevalence is largely underestimated, with only about 60% of DM patients being properly diagnosed and registered, while estimates of prediabetes prevalence among Croatian adult population remain unknown (*5*). Hereby we hypothesized that, equally to other countries, there is a substantial rate of adults with prediabetes and unrecognized DM who could be accurately identified using the HbA_1c_ method certified by the National Glycohemoglobin Standardization Program (NGSP) and traceable to the Diabetes Control and Complications Trial reference assay, as recommended by ADA (*2*). Haemoglobin A1c testing does not require fasting and blood sampling does not have to be limited to a specific time of the day, making it an especially convenient screening tool for the population presenting at the Emergency Department (ED), where these preanalytical requirements cannot be met.

Therefore, the aim of the present study was to perform non-targeted screening of HbA_1c_ concentrations in patients without known DM who underwent any type of laboratory testing at the ED or primary care, and to estimate the prevalence of both prediabetes and undiagnosed DM according to the ADA criteria (*2*).

## Materials and methods

### Subjects and study design

This multi-center, cross-sectional study performed from January to July 2021 included 5527 patients aged 40 to 70 years who were admitted to the ED or had a primary care check-up for any reason, and during their visit underwent routine laboratory diagnostics that included determination of complete blood count. After inspection of patient’s medical documentation, those with confirmed diagnosis of DM or other known disorders of glucose metabolism were excluded. The study was performed in six Croatian public hospitals which geographically covered the majority of the country: University Hospital Center (UHC) Zagreb, Sestre milosrdnice UHC, General Hospital (GH) Šibenik, UHC Split, GH ‘’Dr. Josip Benčević’’ Slavonski Brod and GH Varaždin. Laboratory analyses of HbA_1c_ were performed in medical laboratories within each respective institution, utilizing the method and reagents from the same manufacturer. The obtained HbA_1c_ results were interpreted according to the ADA diagnostic criteria, *i.e.* HbA_1c_ between 39-47 mmol/mol indicate prediabetes while values equal or above 48 mmol/mol were considered as preliminary manifested DM (*2*). Prevalence was calculated as the number of patients with HbA_1c_ concentrations within the defined categories, divided by the total number of included patients. Patients were further divided into three age-stratified groups, *i.e.* from 40 to 49, 50 to 59 and 60 to 70 years.

The study was conducted according to the principles of the Declaration of Helsinki and each institution provided ethical approval from the local Ethics Committee, together with an appropriate patient’s informed consent: UHC Zagreb (8.1-21/14-2, 02/21 AG), Sestre milosrdnice UHC (003-06/21-03/004), GH Šibenik (01-3893/1-21), UHC Split (500-03/21-01/58), GH ‘’Dr. Josip Benčević’’ Slavonski Brod (04000000/21-35) and GH Varaždin (02/1-91/103-2021).

### Methods

Haemoglobin A1c was determined in leftover whole blood samples collected in 3 mL tripotassium ethylenediaminetetraacetic acid tubes produced by either Greiner Bio-one, (Kremsmunster, Austria) or Becton Dickinson, (New Jersey, USA).Blood sampling was part of the routine laboratory work-up at the ED or primary care blood collection points and was not necessarily performed in the fasting state or limited to a specific time of the day. The analyses were performed using the Hemoglobin A1c Reagent Kit (Abbott Laboratories, Chicago, USA) on either Alinity c or Architect c-series analyser (Abbott Laboratories, Chicago, USA). This is an enzymatic method that measures the glycosylated N-terminal fructosyl dipeptides (fructosyl-VH) of the ß-chain of HbA_1c_ after erythrocyte lysis. Total haemoglobin is measured photometrically after its conversion to a stable methaemoglobin azide, while HbA_1c_ as a result of fructosyl-VH oxidation. The final result is expressed in International Federation of Clinical Chemistry units (mmol/mol) as well as National Glycohemoglobin Standardization Program percentage units. Samples were stored at 4 °C and analysed within 24 hours from blood draw, as recommended by the manufacturer.

### Statistical analysis

Shapiro-Wilk test was used for assessment of data normality, while differences in distribution of study participants according to HbA_1c_ categories between settings as well as according to age were assessed with Chi-square test, P < 0.05 was considered statistically significant. Data was processed in Microsoft Excel (Microsoft, Washington, USA) while statistical analysis was performed using MedCalc, v.19.5.2 (MedCalc, Ostend, Belgium).

## Results

Of the initially enrolled 5527 patients, 435 were excluded due to known diagnosis of DM or other disorders of glucose metabolism. Therefore, a total of 5092 participants (median age 57, from 40 to 70 years) were included in prevalence estimation, of whom 2837 (56%) were males. A total of 882 (17.3%) patients had HbA_1c_ values between 39 and 47 mmol/mol, being therefore classified as prediabetes. There were 214 (4.2%) patients with HbA_1c_ ≥ 48 mmol/mol, indicating undiagnosed DM. Prediabetes prevalence ranged from 14.2% to 20.5% among settings, the lowest being in two settings on the Mediterranean coast. Furthermore, undiagnosed DM prevalence ranged from 3.3% to 7.3%. A statistically significant difference (P < 0.001) in distribution of patients according to HbA_1c_ categories among included hospital settings was obtained. Detailed demographic data and the distribution of patients according to HbA_1c_ concentrations *per* each hospital setting and in total are presented in Table 1.

**Table 1:** Participants’ demographic data and distribution according to HbA_1c_ concentrations

	**UHC Zagreb**	**Sestre milosrdnice UHC**	**GH Šibenik**	**UHC Split**	**GH Slavonski Brod**	**GH Varaždin**	**Total**
**Setting**	**ED**	**ED**	**Primary care**	**ED**	**ED**	**ED**
Initial cohort, N	1364	1298	1180	399	332	954	5527
Excluded patients, N (%)	172 (12.6)	145 (11.2)	0*	0*	15 (4.5)	103 (10.8)	435 (7.9)
Patients included in prevalence estimation	1192	1153	1180	399	317	851	5092
Male, N (%)	644 (54)	603 (52)	763 (65)	245 (61)	154 (49)	428 (50)	2837 (56)
Age, years	58 (41 to 70)	58 (40 to 70)	54 (40 to 70)	57 (40 to 70)	57 (40 to 70)	57 (40 to 70)	57 (40 to 70)
HbA_1c_ (mmol/mol)	36 (32–38)	36 (33–39)	34 (32–37)	34 (31–38)	36 (32–39)	33 (31–37)	34 (32–38)
HbA_1c_ < 39 mmol/mol, N (%)	912 (76.5)	864 (74.9)	974 (82.5)	318 (79.7)	237 (74.8)	691 (81.2)	3996 (78.5)
HbA_1c_ 39-47 mmol/mol, N (%)	231 (19.4)	236 (20.5)	167 (14.2)	60 (15.0)	57 (18.0)	131 (15.4)	882 (17.3)
HbA_1c_ ≥ 48 mmol/mol, N (%)	49 (4.1)	53 (4.6)	39 (3.3)	21 (5.3)	23 (7.3)	29 (3.4)	214 (4.2)
Age is presented as median (range). HbA_1c_ - haemoglobin A1c. UHC - university hospital center. GH - general hospital. ED - emergency department. *Excluded prior to laboratory analysis, not recorded.							

Age-stratified analysis of patients’ distribution according to HbA_1c_ concentration yielded a statistically significant difference between age-specific groups (P < 0.001), as shown in Table 2. Prediabetes and undiagnosed DM prevalence increased with age, being 25.4% and 5.8%, respectively, in the group of patients aged 60 to 70 years.

**Table 2:** Age-stratified distribution of study participants according to HbA_1c_ concentrations

**Age group (years),** **N**	**HbA_1c_ < 39 mmol/mol,** **N (%)**	**HbA_1c_ 39 – 47 mmol/mol,** **N (%)**	**HbA_1c_ ≥ 48 mmol/mol, N (%)**	**P**
40–49 (N = 1361)	1264 (92.9)	72 (5.3)	25 (1.8)	< 0.001
50–59 (N = 1673)	1315 (78.6)	288 (17.2)	70 (4.2)	
60–70 (N = 2058)	1417 (68.8)	522 (25.4)	119 (5.8)	
HbA_1c_ - haemoglobin A1c. P < 0.05 was considered statistically significant.				

## Discussion

The present study used HbA_1c_ as a screening tool for prediabetes and unrecognized DM in geographically dispersed adult Croatian population. This approach led to identification of prediabetes in overall 17.3% of the included participants and previously undiagnosed DM in 4.2%, with some clear differences among regions.

Prediabetes prevalence in our study can be compared to the 22.3% overall prevalence of impaired glucose regulation in developed European countries based on either IGT or impaired fasting glucose (IFG) levels criteria (*6*). Furthermore, the International Diabetes Federation official annual data based on IGT results report the global prevalence of 7.3%, with significant heterogeneity worldwide, being 5.5% in Europe and the highest in North America (15.4%) (*4*). However, when either fasting glucose or HbA_1c_ concentrations were applied as criteria, prediabetes prevalence in American adults was estimated to be 34.5% (*7*). Such large differences in prevalence estimations are related to the use of different diagnostic criteria and tests, but also depend on the characteristics and lifestyle of the included population (*4*). In our study, lower prediabetes prevalence, *i.e.* 14.2% and 15.0%, were obtained for the two settings located at the Mediterranean coast compared to those in the continental part of Croatia. This finding might be suggestive of the beneficial effects of the Mediterranean diet on glycaemic control and the overall health status (*8*). Accordingly, awareness about the benefits of preventive lifestyle habits should be more actively promoted, including physical activity, lower calories intake and smoking cessation (*2*, *7*). Additionally, prediabetic patients should be tested yearly to prevent progression to DM, while those with unrecognized DM revealed by HbA_1c_ above 48 mmol/mol require immediate further diagnostic evaluation and medical treatment (*2*). The finding that 4.2% of our patients had previously unknown DM supports the assumption that between one half and one third of people with T2DM are undiagnosed (*2*, *5*). Expectedly, age-stratified analysis showed that prevalence of prediabetes and undiagnosed DM increases with age, further supporting the need for timely screening. Only early risk recognition for developing DM through screening of asymptomatic patients and timely introduction of preventive interventions could supress this continuously increasing trend (*2*, *7*). Although screening based on HbA_1c_ determination is known to yield somewhat higher prediabetes prevalence, and its measurement can be affected by several genetic alterations, erythropoiesis-related conditions and other chronic diseases, it is still the most convenient test for screening purposes. The main advantage is that it does not require fasting as FPG or oGTT, and is less prone to preanalytical errors (*2*, *6*, *7*, *9*). In addition, HbA_1c_ results are not affected by acute illness and acute stress response, making it a valuable tool for screening in the ED. Importantly, elevated HbA_1c_ concentrations were shown to be better predictors of progression to DM than FPG or oGTT, making it a valuable tool for initial identification of patients with impaired glycaemic control (*2*, *7*, *10*).

This study has some limitations. Firstly, it provides only crude estimation of prediabetes and undiagnosed DM prevalence based on single HbA_1c_ determination. However, since the study enrolled mainly patients admitted to the ED, HbA_1c_ determination was the only feasible method as it does not require any specific patient preparation and can be conducted from the existing routine haematological sample. Secondly, it might be loosely speculated that significant proportion of patients presenting at the ED have a higher incidence of risk factors, and therefore higher susceptibility to prediabetes and DM, possibly causing overestimation of the obtained results. Patients presenting at the ED are also expected to be more affected by chronic diseases that might alter HbA1_c_ results. In addition, the specific period of patient enrolment from January to July might have influenced the spectrum of emergencies and therefore possibly enhanced patient selection bias. Finally, we are not aware of the presence of possible rare haemoglobin variants or any other biological confounders of HbA_1c_, *e.g.* end-stage renal failure, liver failure or iron deficiency anaemia that could have affected the final HbA_1c_ results.

In conclusion, as a first study to estimate prediabetes and DM prevalence based on HbA_1c_ concentrations in a large, geographically dispersed sample of Croatian adult population, it identified underlying impairment of glucose regulation in one out of five patients, with a significant number of patients with already overt DM who require prompt medical advice or treatment. Although the patient population admitted to the ED does not completely resemble the characteristics of the general population, this study confirms the potential of large-scale HbA_1c_-based screening for timely identification of adults with prediabetes, as well as undiagnosed DM. The results obtained herein should raise awareness about this widespread healthcare problem and serve as a starting point for further steps directed towards promotion of preventive measures for DM in Croatia.
